# Biomechanical Analysis of Cycle-Tempo Effects on Motor Control Among Jump Rope Elites

**DOI:** 10.3390/bioengineering12020162

**Published:** 2025-02-08

**Authors:** Qi Zhou, Yufeng Liu, Jianguo Kang, Xiuping Wang, Kai Zhang, Gongbing Shan

**Affiliations:** 1 Department of Physical Education, Xinzhou Teachers’ University, Xinzhou 034000, China; zhouqi@xztu.edu.cn (Q.Z.); tyxkjg@xztu.edu.cn (J.K.); wangxp@xztu.edu.cn (X.W.); xzsyzk@xztu.edu.cn (K.Z.); 2 Biomechanics Lab., Faculty of Arts & Science, University of Lethbridge, Lethbridge, AB T1K 3M4, Canada

**Keywords:** biomechanical modeling, 3D motion analysis, rope trajectory, acceleration characteristics, contact time

## Abstract

Jump rope is a widely applied basic training technique in various sports, yet it is understudied biomechanically. This study investigates the impact of cycle-tempo-induced motor control changes in elite jump rope athletes, addressing the biomechanical gap of cyclic skill control. The hypothesis posited two accelerations per jump cycle—one in front of and one behind the body—and anticipated that increased cycle frequency would alter the distribution of acceleration time within a cycle. Using 3D motion capture technology, 12 young elite jump rope athletes were analyzed at 100, 140, and 180 revolutions per minute (rpm). The kinematic parameters obtained confirmed the presence of two distinct accelerations per cycle. As tempo increased, the percentage of rear acceleration time increased from 9.58% at 100 rpm to 17.42% at 180 rpm, while front acceleration time decreased from 39.03% at 100 rpm to 31.40% at 180 rpm, along with peak velocities increasing from 12.94 m/s at 100 rpm to 22.74 m/s at 180 rpm significantly (*p* < 0.01). Rope trajectory analysis indicated a consistent movement pattern across tempos, primarily in the sagittal plane. Variations in skill control revealed shorter contact phases, decreasing from 61.53% at 100 rpm to 48.25% at 180 rpm, as well as a reduced vertical range of motion for the center of gravity (from 0.15 body height at 100 rpm to 0.06 body height at 180 rpm) and feet (from 0.05 body height at 100 rpm to 0.03 body height at 180 rpm) (*p* < 0.05). Significant reductions were also observed in the flexion/extension range of motion for the hip (from 22.31° at 100 rpm to 3.47° at 180 rpm), knee (from 49.31° at 100 rpm to 9.35° at 180 rpm), and ankle (from 52.99° at 100 rpm to 21.41° at 180 rpm) (*p* < 0.01). These findings enhance the understanding of motor control adaptations to different tempos and have practical implications for developing coaching programs aimed at optimizing performance, stability, and efficiency in jump rope training.

## 1. Introduction

Jumping rope has long been widely applied as a versatile and practical method for enhancing athletic conditioning, balance, and coordination across various sports disciplines [1,2,3]. Its simplicity belies its effectiveness, making it a staple in training regimes worldwide [1,2,3]. Athletes, from amateur enthusiasts to elite professionals, incorporate jumping rope into their workouts to improve agility, endurance, and overall performance [4,5,6,7]. However, despite its extensive use, a significant gap exists in our understanding of how variations in jumping speed influence motor control patterns among athletes. A search conducted in November 2023 using the keywords “rope jump + speed-dependent motor control” in the Web of Science database returned no relevant results, highlighting a gap in research on this topic in relation to training practices.

The speed of rope jumping, normally quantified by revolutions per minute (rpm) [8,9], plays a pivotal role in performance. Athletes aiming to increase rpm encounter heightened physical demands [9,10,11], necessitating precise motor control to sustain rhythm, balance, and coordination. It is widely hypothesized that as jumping speed rises, distinct changes in motor control patterns occur, impacting both technique and efficiency.

The scarcity of published articles investigating speed-dependent motor control pattern changes in jumping rope underscores the significant research gap. While numerous studies have explored the benefits of jumping rope on athletic attributes such as jump strength, running speed, endurance, and agility [12,13,14], none have specifically addressed how increasing speed influences motor control strategies. This dearth of scientific evidence presents an unprecedented opportunity for pioneering research in the field.

Speed, as a temporal factor, is crucial for optimizing motor control in complex sports skills [15,16,17,18]. Therefore, understanding the nuances of motor control adaptations to varying jumping speeds is essential for optimizing training methods and enhancing athlete performance. By elucidating the relationship between speed and motor control, researchers can inform evidence-based coaching strategies tailored to the specific demands of jumping rope activities. Furthermore, insights gained from this research have the potential to extend beyond the realm of jumping rope to inform training practices in other sports and physical activities where speed and coordination are integral components.

To ensure reliable motor control pattern analysis, the quality of subjects is paramount [19,20,21]. Professional jumping rope athletes, distinguished by years of rigorous training and competition experience, offer an ideal cohort for examining speed-dependent motor control pattern changes. Their refined motor skills and consistent performance levels provide a stable baseline against which potential alterations in control patterns can be discerned. By studying this population, reliable results can be obtained, elucidating nuanced adaptations in motor control strategies at varying jumping speeds.

Rope jumping entails a cyclic movement. However, due to the absence of biomechanical quantification of this cyclic skill control, the acceleration characteristics within a cycle remain unclear. Drawing from practitioners’ experiences, we hypothesize the presence of two accelerations in each jump cycle—one occurring in front of the body and the other behind the body. It is anticipated that the total time for rope movement within a complete cycle will decrease with increasing cycle frequency, along with both accelerations. Nonetheless, practitioners may find it intriguing to ascertain the relative change in control within a cycle, particularly the percentage change of acceleration times in a cycle. Building upon practitioners’ experiences, we propose the second hypothesis for the current study: as cycle frequency increases, the percentage of acceleration time in front of the body will decrease, while the percentage behind the body will increase. Consequently, the primary aim of this investigation is to examine the alterations in speed-dependent motor control patterns among elite jumping rope athletes. Our objective is to elucidate the relationship between jumping speed variations and motor control alterations, thereby addressing the existing gap in coaching practice. The findings from this study carry practical implications for the development of targeted training programs designed to optimize athletic conditioning, balance, and coordination.

In short, this study aims to contribute novel insights into athletic training by examining the impact of rope tempo on motor control patterns among elite jumping rope athletes. By bridging the gap between theory and practice, we aspire to inform evidence-based coaching strategies that optimize athletic performance and enhance the overall effectiveness of training programs.

## 2. Materials and Methods

### 2.1. Subjects

As elaborated in the introduction, a central goal of this study is to collect biomechanically appropriate kinematic data while minimizing any influence of the testing process. Consequently, individuals with stable skill control were required. Elite athletes, characterized by their well-trained motor-control stability resulting from extensive training, were deemed ideal candidates. It is important to note that recruiting elite participants for this type of study is particularly challenging due to their limited availability and other practical constraints. As a result, sample sizes in similar studies are typically small, ranging from 2 to 9 individuals [21]. In this pilot study, we made our best efforts to recruit as many elite participants as possible, resulting in a sample size of 12 male Chinese participants. Table 1 presents an overview of their physical attributes and relevant experiences. All participants had not experienced any sports injuries within the past year and were in good physical and mental condition during testing. The test protocol was thoroughly scrutinized and approved by the host university’s ethics committee. Prior to the commencement of the study, participants were briefed on the testing procedures, signed consent forms, and voluntarily participated in the data collection process.

### 2.2. Test Protocol

From a technical and performance standpoint, it is observed that the majority of proficient individuals with developed jumping rope skills maintain rope frequencies ranging between 100 rpm and 180 rpm [23,24]. Consequently, this study opts to commence at 100 rpm as a baseline, considering that jumpers at this frequency already demonstrate continuous, relatively stable, and refined rope-swinging techniques [23]. The tempo within the range of 92–100 rpm is regarded as optimal for rope jumping from an injury prevention standpoint, as it minimizes joint stress, particularly on the knees [11]. Beyond 100 rpm, as the jumping cycle accelerates, there is an increase in physical demands [11]. Notably, a frequency of 140 rpm serves as a critical threshold that jumpers and coaches should heed [1]. When assessing the physical demands of rope jumping, factors such as physiological readiness, coordination efficiency, and rope cycle need to be taken into account. Specifically, when the frequency surpasses 140 rpm, there are significant disparities in heart rate and oxygen consumption compared to frequencies below 140 rpm [1]; Quirk and Sinning [9] similarly observed notable increases in these parameters between 140 rpm and 160 rpm, with no significant distinctions between 120 rpm and 140 rpm. Additionally, these differences are absent across frequencies of 66, 84, 102, 120, and 132 [10]. Experienced jumpers typically adhere to a frequency of 138 rpm, with 140 rpm marking the lower threshold of the aerobic training frequency range for rope jumping [25]. In the majority of regions in China, approximately 180 rpm is regarded as the benchmark for middle school physical education rope jumping assessments. Achieving a speed of 180 rpm or higher is indicative of exceptional physical prowess, particularly concerning balance, reaction time, and coordination among young adolescents in China [26]. Moreover, a speed of 180 rpm meets the criteria for activating anaerobic energy systems through rope training [25]. Therefore, this study incorporates three representative frequencies, namely 100 rpm, 140 rpm, and 180 rpm.

The participants were guided through the three test tempos by a metronome, ensuring compliance with the required rhythms for each test condition. Before the commencement of the testing session, participants engaged in a ten-minute self-warm-up routine. Subsequently, participants executed three sets of rope jumping at frequencies of 100 rpm, 140 rpm, and 180 rpm, each lasting for 8 s. A 3 min rest interval was provided between frequency adjustments to allow participants to exert consistent physical effort (i.e., without experiencing fatigue) in completing all experimental tasks.

### 2.3. Three-Dimensional Motion Capture and Biomechanical Modeling

**Figure 1:**
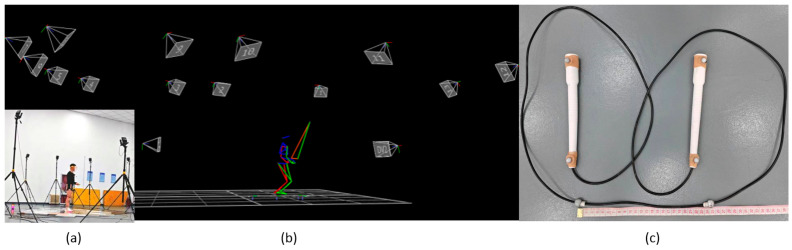
The setup of 3D motion capture: (**a**) camera setup, (**b**) an exemplary illustration of a captured instant of the 3D jumping rope with camera positions, and (**c**) the marker configuration on the rope.

To capture the movement involved in jumping rope, a 3D motion-capture system with 13 high-speed cameras was utilized, incorporating 45 reflective soft markers (diameter = 9 mm), comprising 39 markers positioned on the body and 6 on the rope. Additionally, 2 camcorders (DV1 and DV2) were synchronized with the 3D motion-capture system to supply video references (Figure 1).

The motion capture process employed a 13-high-speed-camera (200 frames/s) motion capture system (VICON MX40, Oxford Metrics Ltd., Oxford, UK). Extensive calibration procedures were conducted to minimize calibration residuals, following the guidelines provided by the manufacturer, resulting in an accuracy of <1 mm. Additionally, two CASIO video camcorders (100 Hz) synchronized with the VICON system were used to record all test trials, facilitating rapid visual assessments of trial quality.

The rope selected was constructed from PVC material designed for speed ropes, as it maximizes rope aerodynamics and can readily achieve speeds of 5 revolutions per second (i.e., 300 rpm) [25,27]. To standardize variables, the same jump rope was utilized for all participants, with a length extending from the feet to the upper chest. This length ensures optimal comfort for advanced jumpers without compromising body posture and technique during jumps, with rotational speeds of up to 240 rpm [25,27]. Two reflective soft markers were positioned in the middle of the jump rope, spaced 25 cm apart (Figure 1c), to prevent interference with the feet during jumps. Placing two reflective markers in the middle of the rope serves two purposes: firstly, to mitigate the impact of rope midpoint rebound on data integrity, and secondly, to ensure comprehensive and accurate data collection of rope revolutions. Additionally, four markers, two on each handle, were utilized to delineate the rope during jumping.

The strategic placement of 39 body markers facilitated the construction of a 15-segment biomechanical model [28,29], encompassing segments such as the head, upper and lower trunk, upper and lower arms, hands, thighs, shanks, and feet. The 15-segment model has been validated for assessing numerous complex sports skills in prior research [30,31,32,33]. The precise positioning of the 39 markers was conducted at specific anatomical landmarks on the subjects, including regions such as the temporal and posterior head regions, sternal end of the clavicle, xiphoid process of the sternum, C7 and T10 vertebrae, scapulae, iliac regions, acromion processes, epicondyles, and styloid processes of the radii and ulnae, among others. The use of 13 cameras and small markers provided subjects with substantial freedom of movement during motion capture, allowing them to closely replicate their trained “motor control style” to obtain their control patterns at different tempos.

### 2.4. Data Processing, Parameter Selection, and Statistical Analysis

To ensure stable control, we selected 5 revolutions near the midpoint of each 8 s data collection. The beginning of a revolution was defined as the midpoint of the rope at its lowest point in the vertical direction. This process resulted in a total of 180 revolutions of 3D data (3 tempos × 12 subjects × 5 revolutions = 180) for analysis. The raw motion capture data were processed using a five-point smoothing filter within the VICON software (Nexus 2.8.1, VICON Motion Systems, Oxford Metrics Ltd., Oxford, UK). This processing resulted in a dataset containing the 3D coordinates of 39 markers. These processed coordinates were then used to create a 15-segment biomechanical model [31,32] for analyzing the kinematics of rope jumping.

Biomechanical models provide a wide array of kinematic parameters, such as joint angles, velocities, accelerations, and more, derived from the 3D coordinates of captured markers on athletes to unveil control insights into complex sports skills [32,33,34]. However, it is imperative to select relevant data for the clear and concise communication of research findings. Focused reporting and discussion are essential principles in research writing [35,36], ensuring that the study’s aims remain central. Therefore, researchers must carefully choose parameters aligned with their paper’s focus to maintain coherence. Given the study’s objective of quantifying the influence of rope revolution changes on control pattern variations, the primary outcomes focused on the velocity of the rope midpoint, rope handle, and big toe, as well as the timely coordination among these parameters, joint parameters, and center of gravity (COG) [16,37]. The secondary outcomes aimed to establish motor control patterns at different rope tempos, providing biomechanical insights for coaching practices.

The 3D coordinates of the rope midpoint were determined by averaging the coordinates of the two markers on the rope, each located 12.5 cm from the midpoint (Figure 1c). Speed and acceleration changes over time of the rope midpoint were calculated using frame-by-frame calculations of the average values in Microsoft Excel. Similarly, the averages of rope-handle markers and big toe markers were used to quantify the 3D excursions of the rope-handle point (center of cycle movement) and feet (dynamic distance to the ground), respectively. Additionally, two more calculated parameters, ROM (range of motion, determined by max–min) and % of a cycle time, were applied for kinematic analysis. This included the above parameters and the timely excursion of joint angles and COG obtained from the 15-segmental model quantification. With this approach, the potential control pattern changes induced by the tested tempos were quantitatively analyzed. To provide user-friendly results (e.g., minimizing the influence of body height in motor learning) for practitioners, normalization (% of body height) was applied to length-related parameters.

Statistical analysis was conducted on the data collected. Descriptive statistics, such as mean and standard deviations (mean ± SD), were calculated to demonstrate parameter characteristics. The normality of the data was evaluated using the Shapiro–Wilk test. If normality was confirmed, a one-way ANOVA was performed to identify changes in control patterns caused by variations in rope revolution frequency. All statistical analyses were carried out using IBM SPSS Statistics 23 (IBM Japan, Tokyo, Japan), with a significance level set at *p* = 0.05.

## 3. Results

### 3.1. Rope Trajectory

**Figure 2:**
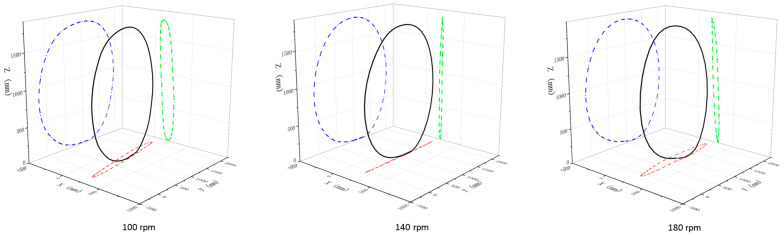
A 3D cycle trajectory (black) of the rope midpoint and its projections on 3 analysis planes (blue: sagittal plane, green: frontal plane, and red: transverse plane).

The rope cycle movement is quantified by the rope-handle point (center of cycle movement) and the rope midpoint (rope trajectory). The analysis shows that the rope trajectory is primarily in the sagittal plane (Figure 2), and this movement pattern is not influenced by the three tempos, as there were no significant differences among the rope-handle point and rope midpoint during cycle movement (*p* > 0.05).

### 3.2. Rope Control Variation

**Figure 3:**
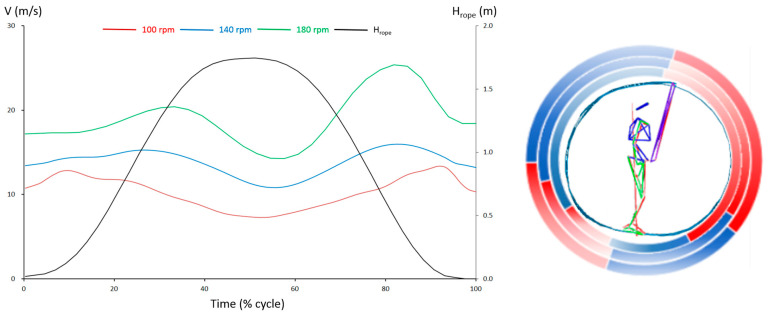
Variations in rope control within a cycle influenced by the three cycle tempos. The (**left panel**) illustrates the dynamic changes in rope-midpoint velocity during a cycle (H_rope_: height of rope midpoint). Both the changes in absolute values and the time shifting of peak values are associated with variations in cycle tempo. The (**right panel**) depicts the spatial zones corresponding to the acceleration (red) and deceleration (blue) phases within a cycle. These relative phases are also influenced by the three cycle tempos (from inside to outside: 100 rpm, 140 rpm, and 180 rpm).

As hypothesized, each rope cycle includes two accelerations, evidenced by two peaks in the velocity change-over-time curve for each cycle (Figure 3, left). The first peak occurs behind the body, termed rear-body acceleration (rear acceleration), and the second peak occurs in front of the body, termed front-body acceleration (front acceleration).

As rope tempo increases, the cycle time decreases significantly from 0.6 ± 0.01 s at 100 rpm to 0.43 ± 0.01 s at 140 rpm, reaching 0.33 ± 0.01 s at 180 rpm (*p* < 0.01). Correspondingly, the two peak velocities increase significantly from 13.39 ± 1.11 m/s at 100 rpm to 16.32 ± 1.13 m/s at 140 rpm, reaching 20.62 ± 0.73 m/s at 180 rpm (*p* < 0.01) for rear max velocity, and from 12.94 ± 1.20 m/s at 100 rpm to 16.88 ± 1.56 m/s at 140 rpm, reaching 22.74 ± 1.68 m/s at 180 rpm (*p* < 0.01) for front max velocity (Table 2 and Table 3).

Regarding the relative time (% of a cycle) of the peak velocities, the results clearly show that the two peaks are close to the lowest point of the rope midpoint (Figure 3, left, and Table 2). However, significant differences (*p* < 0.01) are found only between 100 and 140 rpm, as well as between 100 and 180 rpm, not between 140 and 180 rpm (*p* > 0.05).

The acceleration–time characteristics within a cycle reveal the reasons for the velocity variations influenced by rope tempos (Table 4 and Table 5). In terms of magnitude, there are no significant differences among the rear accelerations (*p* > 0.05). However, the front acceleration increases significantly from 25.17 ± 13.73 m/s^2^ at 100 rpm to 43.76 ± 15.70 m/s^2^ at 140 rpm, reaching 86.66 ± 29.85 m/s^2^ at 180 rpm (*p* < 0.01). Regarding timing, while there is no significant difference in the relative total acceleration time among the three tempos (*p* > 0.05), the rear acceleration time (9.58% ± 2.94%) at 100 rpm is significantly shorter than at 140 rpm (16.47% ± 5.38%) and 180 rpm (17.42% ± 4.68) (*p* < 0.01). Conversely, the front acceleration time (39.03% ± 8.63%) at 100 rpm is significantly longer than at 140 rpm (31.40% ± 5.57%) and 180 rpm (31.57% ± 5.70) (*p* < 0.01). There are no significant differences between 140 rpm and 180 rpm (*p* > 0.05). Figure 3 (left) illustrates these acceleration characteristics across the three tempos.

### 3.3. Skill Control Variation

**Figure 4:**
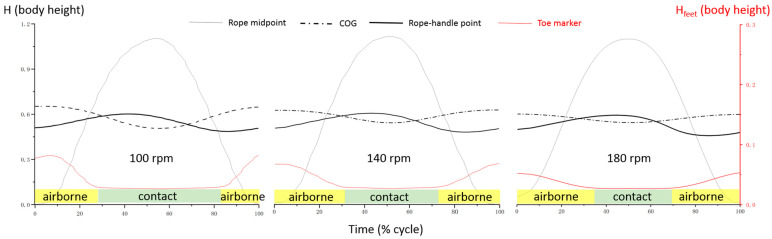
A typical position change over time for selected key markers. H (body height): height, H_feet_ (body height): feet height.

Figure 4 illustrates a typical example of skill control variation. As the rope tempo increases, the contact phase shortens, and the ROM (range of motion) of the COG (center of gravity) and feet in the vertical direction decrease. The movement patterns of the rope midpoint and rope-handle point remain unaffected by the different tempos.

**Figure 5:**
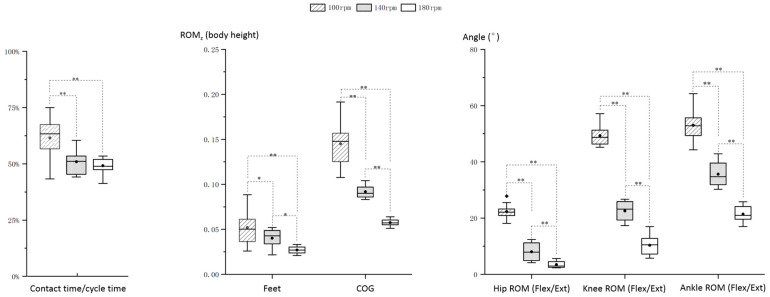
Selected skill control parameters significantly influenced by rope tempos. *: *p* < 0.05, **: *p* < 0.01, Flex/Ext: flexion/extension.

Statistical analysis reveals cycle-tempo-induced motor control changes, as shown in Figure 5. As the tempo increases, the contact time at 100 rpm (61.53% ± 9.03%) is significantly longer (*p* < 0.01) than at 140 rpm (50.97% ± 5.65%) and 180 rpm (48.25% ± 5.08%). There are no significant differences between 140 rpm and 180 rpm (*p* > 0.05) (Figure 5, left).

Regarding the ROM of feet and COG height (% of body height), both continuously decrease as tempo increases. The feet ROM decreases significantly from 0.05 ± 0.02 (body height) at 100 rpm to 0.04 ± 0.01 at 140 rpm, reaching 0.03 ± 0.00 at 180 rpm (*p* < 0.05). Similarly, the COG ROM decreases significantly from 0.15 ± 0.03 (% of body height) at 100 rpm to 0.09 ± 0.01 at 140 rpm, reaching 0.06 ± 0.00 at 180 rpm (*p* < 0.01) (Figure 5, middle).

The significant joint ROM changes are only found in lower limb joint flexion/extension. As tempo increased, the hip ROM decreased significantly from 22.31° ± 2.56° at 100 rpm to 8.00° ± 3.18° at 140 rpm, reaching 3.47° ± 1.22° at 180 rpm (*p* < 0.05). Similarly, the knee ROM decreased significantly from 49.31° ± 13.81° at 100 rpm to 22.55° ± 3.49° at 140 rpm, reaching 9.35° ± 3.37° at 180 rpm (*p* < 0.01) and the ankle ROM decreased significantly from 52.99° ± 5.60° at 100 rpm to 35.63° ± 4.16° at 140 rpm, reaching 21.41° ± 2.90° at 180 rpm (*p* < 0.01).

## 4. Discussion

The present study aimed to explore the impact of cycle-tempo-induced motor control changes in elite jump rope athletes. By examining the biomechanical aspects of rope jumping at various tempos, this research sought to fill a gap in the understanding of cyclic skill control in this athletic activity. The findings provide valuable insights into the relationship between jumping speed and motor control, offering practical implications for training and coaching practices.

### 4.1. Key Findings and Hypotheses Evaluation

The study’s first hypothesis posited that each jump cycle includes two accelerations, one occurring in front of the body and the other behind it. The results have confirmed this hypothesis, revealing distinct velocity peaks in the velocity change-over-time curve for each cycle. These findings align with practitioners’ observations, supporting the notion that the rope’s movement entails significant accelerations at specific points within the cycle [25,38]. However, a novel aspect of this preliminary biomechanical study is that the two peak velocities are close to the lowest point of the rope midpoint, indicating that rear and front accelerations occur near the rope–foot intersection. This result highlights the unique timing characteristics of rope control, which should be emphasized in the skill learning and training process to increase learning/training efficiency [37]. This insight enhances the understanding of the skill control demands of jump rope, highlighting the importance of wrist control and its role in hand–foot coordination for optimizing performance.

The second hypothesis suggested that with increasing cycle frequency, the percentage of acceleration time in front of the body would decrease while the percentage behind the body would increase. The results have partially supported this hypothesis. While the relative total acceleration time did not vary significantly across tempos, the distribution of acceleration time between front and rear periods showed a two-stage change. Specifically, rear acceleration time increased significantly between 100 rpm and 140 rpm (*p* = 0.00) and remained relatively unchanged between 140 rpm and 180 rpm (*p* > 0.05). Similarly, front acceleration time decreased significantly between 100 rpm and 140 rpm (*p* = 0.00) and remained relatively unchanged between 140 rpm and 180 rpm (*p* > 0.05). This two-stage increase might suggest the existence of a critical tempo, beyond which the rear–front acceleration time ratio stabilizes at approximately 1:2. These findings imply that targeted timing control training could enhance training efficiency [39,40]. Further studies with larger samples are needed to validate these results.

### 4.2. Rope Trajectory and Training-Induced Control Adaptation

The analysis of rope trajectory indicated that the rope’s movement predominantly occurs in the sagittal plane, with no significant differences among the three tempos. This consistency in movement pattern underscores the stability of the well-trained athletes’ technique across varying speeds. The stability of the rope trajectory despite tempo changes suggests that elite athletes possess a high level of rope control that allows them to maintain consistent patterns under different tempo conditions. The results may indicate that rope jumping training helps trainees improve timely coordination between upper and lower limbs, especially between hands and feet. These preliminary biomechanical results provide direct evidence to support empirical findings from previous studies that suggest rope jumping improves dynamic coordination under various age and physical conditions [38,41,42,43,44].

Furthermore, the biomechanical analysis revealed that as rope tempos increased, the cycle time decreased significantly (*p* = 0.00), accompanied by a significant increase in peak velocities (*p* = 0.00) for both rear and front acceleration phases. This inverse relationship between cycle time and peak velocities indicates that higher tempos demand more rapid and forceful movements, exemplifying the basic physics principle: power = force × velocity in sport applications [45]. Specifically, the greater increase in front maximum velocity at higher tempos highlights the importance of increasing power control in this phase, which is crucial for successfully maintaining the rope’s changing rhythm. To improve learning and training efficiency, this aspect should also be emphasized in coaching practice.

A further novel finding of the current study is the extremely low foot height at the rope–foot intersection point, particularly at 180 rpm. On average, this height is only 3% of the athlete’s body height, translating to approximately 5 cm for an athlete who is 1.7 m tall. This observation explains why high tempos often lead to failure in practice, as both the time available for coordination and the allowable height decrease significantly (*p* = 0.00).

Lastly, combining the above results, increasing cycle-rope-tempo training improves timely acceleration control, limb coordination, and power generation and control. All of these characteristics are essential for enhancing athlete agility [46], especially in team sports [47]. From this perspective, this study provides biomechanical evidence explaining why rope jumping training can enhance athletic agility across various sports, as observed in previous studies [6,48,49,50,51]. The findings underscore the value of rope jumping as a training modality for developing the agility crucial for performance in a wide range of athletic disciplines.

### 4.3. Cycle-Tempo-Induced Body Control Pattern Changes

The skill control variation analysis demonstrated that increased rope tempos led to a shorter contact phase and reduced range of motion (ROM) for both the center of gravity (COG) and feet in the vertical direction. These changes suggest that athletes adapt to higher tempos by minimizing vertical displacement, which likely contributes to more efficient energy use and quicker cycle times. The unchanged movement patterns of the rope midpoint and rope-handle point across different tempos further emphasize the athletes’ ability to maintain a consistent rope path while adapting to cycle-tempo variations. This newly unveiled control insight suggests a separation of upper and lower body control: the upper body maintains the consistency of the rope path regardless of rope-tempo variations, while the legs adapt to rope-tempo changes by reducing vertical movement of the feet and COG. This separation indicates the complexity and optimization of motor control developed through varied rope-tempo training, which enhances athletes’ coordination abilities.

Currently, the literature lacks a consensus on the precise definition of joint-locking control. Based on the normal flexion/extension ROM of the hip, knee, and ankle in adult males [52], a cutoff value for joint locking/unlocking can be established at 10°—i.e., if the ROM is less than 10°, the joint is considered locked; otherwise, it is actively controlled. Using this cutoff value for joint locking identification, the ROM results of leg joints revealed new insights into lower limb control patterns: a hip–knee–ankle control strategy at 100 rpm, a knee–ankle control strategy at 140 rpm, and an ankle control strategy at 180 rpm. As rope tempo increases, the body becomes progressively stiffer, with the control pattern transitioning from multi-joint control to ankle-dominant control. This adaptation likely aids in maintaining stability and efficiency by limiting excessive joint motion under high-speed conditions. The results suggest that high-tempo training is beneficial for enhancing ankle power, potentially explaining why rope jumping is frequently utilized in sprinter and agility training across various sports [53,54,55].

### 4.4. Limitations, Practical Implications and Future Research

The current study, akin to numerous scholarly pursuits, presents constraints that necessitate contemplation. Notably, it embodies characteristics typical of a study with a limited sample size, which is susceptible to substantial biases. Small sample sizes are often inevitable in research involving skilled or elite athletes due to the limited availability of such subjects. However, one advantage is the high stability of control patterns developed through targeted training [32]. Typically, studies with small sample sizes in elite sports encompass 2 to 9 qualified subjects [56,57,58,59,60]. Our investigation adheres to this framework, albeit attempts were made to increase the sample size to 12 individuals.

An essential consideration in studies with a limited sample size revolves around whether the research objective emphasizes generalizable inferences or insights closely linked to practical scenarios. Our study is explicitly framed as a case-oriented inquiry, concentrating on furnishing intricate insights rather than deriving broad, generalizable conclusions. The case study methodology facilitates the examination of intricate units comprising numerous variables pertinent to the phenomenon under examination [61]. Grounded in authentic contexts, case studies furnish thorough and nuanced insights that can guide future research initiatives [61].

A further limitation pertains to the study’s exclusive focus on elite males, raising questions regarding the extensibility of the findings to a broader populace. To fortify the reliability and relevance of these findings, forthcoming research should prioritize participant diversity, validating and potentially broadening the insights gleaned from this particular cohort.

The findings of this study have several practical implications for developing training programs aimed at enhancing athletic performance in jump rope. Understanding the specific motor control adjustments required at different tempos can inform evidence-based coaching strategies. Coaches can design targeted training drills that focus on improving acceleration control and optimizing joint ROM to enhance performance at varying speeds.

Future research should build on these findings by investigating the underlying neuromuscular mechanisms that facilitate these motor control adaptations. Additionally, exploring the impact of fatigue on motor control patterns and performance at different tempos could provide further insights into training and competition preparation. Longitudinal studies examining the effects of specific training interventions on motor control and performance in elite jump rope athletes would also be valuable.

## 5. Conclusions

This study offers a comprehensive biomechanical analysis of cycle-tempo-induced motor control changes in elite jump rope athletes. The findings confirm the presence of distinct acceleration phases within each jump cycle and reveal significant adjustments in motor control strategies as tempos increase. These insights enhance the understanding of the biomechanical demands of rope jumping and provide practical guidance for optimizing training and coaching practices. By bridging the gap between theory and practice, this research contributes to the development of evidence-based strategies that can enhance athletic performance and training effectiveness in jump rope.

## Figures and Tables

**Table 1:** Participants’ anthropometrical and training information.

Age (Years)	Height (cm)	Weight (kg)	Training (Years)	Training (Hours/Week)	Level ^1^
20.11 ± 2.4	170.24 ± 2.1	68.2 ± 3.4	4.5 ± 0.5	15.2 ± 2.1	1

^1^ Chinese National Certificate System for Athlete Performance Level. In this system, the performance levels are classified as follows: 1 denotes professional athletes, 2 signifies pre-professional athletes, and 3 refers to individuals who achieve a placement within the top six positions in competitions conducted at the county level or higher [22].

**Table 2:** Average and standard deviation (average ± SD) of the selected time–velocity variables associated with the influences of rope tempos on rope control.

Parameter	100 rpm	140 rpm	180 rpm
Cycle time (s)	0.60 ± 0.01	0.43 ± 0.01	0.33 ± 0.01
Rear V_max_ (m/s)	13.39 ± 1.11	16.32 ± 1.13	20.62 ± 0.73
Time at rear V_max_ (% of a cycle)	13.02 ± 4.20	21.76 ± 6.99	25.60 ± 5.60
Front V_max_ (m/s)	12.94 ± 1.20	16.88 ± 1.56	22.74 ± 1.68
Time at front V_max_ (% of a cycle)	89.17 ± 2.58	83.59 ± 4.77	85.06 ± 4.45

V_max_: max velocity.

**Table 3:** Significance identified by *p*-value obtained from ANOVA with the probability of finding effects obtained from power analysis (*p*-value, probability) associated with the selected time–velocity parameters.

Parameter	100–140 rpm	140–180 rpm	100–180 rpm
Cycle time (s)	0.00, 1.00 **	0.00, 1.00 **	0.00, 1.00 **
Rear V_max_ (m/s)	0.00, 1.00 **	0.00, 1.00 **	0.00, 1.00 **
Time at rear V_max_ (% of a cycle)	0.00, 0.99 **		0.00, 1.00 **
Front V_max_ (m/s)	0.00, 1.00 **	0.00, 1.00 **	0.00, 1.00 **
Time at front V_max_ (% of a cycle)	0.01, 0.86 **		0.01, 0.82 **

V_max_: max velocity, **: highly significant (*p* ≤ 0.01).

**Table 4:** Average and standard deviation (average ± SD) of the selected time–acceleration variables associated with the influences of rope tempos on rope control.

Parameter	100 rpm	140 rpm	180 rpm
Total acceleration time (% of a cycle)	48.61 ± 10.04	47.87 ± 6.97	48.99 ± 5.31
Average rear acceleration (m/s^2^)	40.13 ± 17.18	43.87 ± 22.54	51.75 ± 20.25
Rear acceleration time (% of a cycle)	9.58 ± 2.94	16.47 ± 5.38	17.42 ± 4.68
Average front acceleration (m/s^2^)	25.17 ± 13.73	43.76 ± 15.70	86.66 ± 29.85
Front acceleration time (% of a cycle)	39.03 ± 8.63	31.40 ± 5.57	31.57 ± 5.70

**Table 5:** Significance identified by *p*-value obtained from ANOVA test with the probability of finding effects obtained from power analysis (*p*-value, probability) associated with the selected time–acceleration parameters.

Parameter	100–140 rpm	140–180 rpm	100–180 rpm
Total acceleration time (% of a cycle)			
Average rear acceleration (m/s^2^)			
Rear acceleration time (% of a cycle)	0.00, 0.81 **		0.00, 0.86 **
Average front acceleration (m/s^2^)	0.00, 0.99 **	0.00, 1.00 **	0.00, 1.00 **
Front acceleration time (% of a cycle)	0.00, 0.80 **		0.00, 0.81 **

**: highly significant (*p* ≤ 0.01).

## Data Availability

The data presented in this study are available on request and after appropriate IRB approvals.
